# International Commission on Trichinellosis: Recommendations on post-harvest control of *Trichinella* in food animals

**DOI:** 10.1016/j.fawpar.2019.e00041

**Published:** 2019-02-21

**Authors:** Karsten Noeckler, Edoardo Pozio, Joke van der Giessen, Dolores E. Hill, H. Ray Gamble

**Affiliations:** a Federal Institute for Risk Assessment, Diedersdorfer Weg 1, 12277 Berlin, Germany; b Department of Infectious, Parasitic and Immunomediated Diseases, Istituto Superiore di Sanità, viale Regina Elena 299, 00161 Rome, Italy; c National Institute for Public Health and the Environment (RIVM), Center for Zoonoses and Environmental Microbiology, Antonie van Leeuwenhoeklaan 9, 3721, MA, Bilthoven, Netherlands; d United States Department of Agriculture, Agricultural Research Service, Animal Parasitic Diseases Laboratory, Beltsville, MD 20705, United States of America; e National Academy of Sciences, 500 Fifth Street NW, Washington, DC 20001, United States of America

**Keywords:** *Trichinella*, Trichinellosis, Post-harvest, Food safety

## Abstract

Domestic and wild animals which consume meat are at risk of becoming infected with *Trichinella* and therefore may pose a public health risk. Among domestic livestock, pigs are most commonly associated with *Trichinella* infection, but human outbreaks have also resulted from consumption of horsemeat, wild boar, bear, walrus and other wild animals. For animals that are not produced under controlled management conditions and for wild animals, specific steps should be taken to prevent human exposure to *Trichinella*. These steps include appropriate testing of individual carcasses to identify those that pose a public health risk, post-slaughter processing to inactivate *Trichinella* in meat that might be infected, and education of consumers regarding the need for proper preparation methods for meat that might contain *Trichinella* larvae. The International Commission on Trichinellosis recognizes three (3) acceptable means of treatment to render potentially *Trichinella*-infected meats safe for consumption: 1) cooking, 2) freezing (for meat from domestic pigs), and 3) irradiation. Proper use of these methods is described here, along with specific cautions on use of other methods, including curing and heating with microwaves.

## Highlights

• Domestic and wild animals which consume meat are at risk of *Trichinella* infection.
• Post-harvest inspection methods for *Trichinella* in pigs reduce human disease.
• Heating is acceptable for treatment of meat to inactivate *Trichinella*.
• Freezing is acceptable for the inactivation of *Trichinella* in domestic pork.
• Curing processes must be validated to prove inactivation of *Trichinella*.

## 1 Introduction

Human trichinellosis is acquired by ingestion of uncooked or undercooked meat containing *Trichinella* larvae. Domestic and wild animals which consume meat are at risk of becoming infected and therefore may pose a public health risk. Among domestic livestock, pigs are most commonly associated with *Trichinella* infection. Since 1975, horses have also been recognized as a source of human trichinellosis where horsemeat is consumed raw or not adequately cooked (Pozio et al., 2001). Many types of wild animals can harbor *Trichinella*, and human outbreaks have been attributed to numerous animal species, notably, wild boar, bear, and walrus, among others (Pozio, 2007).

For livestock produced under conditions of controlled management, the risk of exposure to *Trichinella* is negligible. Recommendations for animal production systems that aim to prevent exposure of domestic animals to *Trichinella* are found in the *International Commission on Trichinellosis Recommendations on Pre-Harvest Control of Trichinella in Food Animals* (http://www.trichinellosis.org/Guidelines.html) and in Gamble et al. (2019). For animals that are not produced under controlled management conditions and for wild animals, specific steps should be taken to prevent human exposure to *Trichinella*. These steps include appropriate testing of individual carcasses to identify those that pose a public health risk, post-slaughter processing to inactivate *Trichinella* in meat that might be infected, and education of consumers regarding the need for proper preparation methods for meat that might contain *Trichinella* larvae.

Here, we describe the use of post-harvest testing along with specific recommendations for the use of post-harvest processing methods. Further details on the testing methods can be found in the *ICT Quality Assurance in Digestion Testing Programs for* Trichinella (http://www.trichinellosis.org/Guidelines.html), and in Gajadhar et al. (2019).

## 2 Post-harvest testing methods (individual animal inspection)

Post-harvest inspection methods for *Trichinella* in pigs have been highly effective in reducing human disease resulting from eating raw or improperly cooked pork, and these methods have been used for testing of other food animal species (e.g., horses, farmed dogs and crocodiles) and wild animals as well.

Due to some cultural habits of eating horsemeat without substantial cooking, and given the history of human trichinellosis resulting from eating horsemeat, the ICT recommends testing individual carcasses of horses for *Trichinella* infection following methods described in the *ICT Quality Assurance in Digestion Testing Programs for* Trichinella and in Gajadhar et al. (2019).

Many carnivorous and omnivorous mammals, reptiles and birds can be sources of *Trichinella* infection in humans. Therefore, the ICT recommends that all susceptible wild animals that are intended for human consumption similarly be tested for *Trichinella*.

### 2.1 Direct testing methods

The ICT-recommended method for testing individual animal carcasses is the artificial digestion method for pooled samples, as referenced above. Other descriptions of pooled sample digestion methods can be found in the OIE Manual of Diagnostic Tests and Vaccines for Terrestrial Animals (Chapter 2.1.20) (World Organization for Animal Health – OIE, 2017), European Commission Regulation 2015/1375 (European Commission, 2015) and ISO 18743:2015. Any of the described methods for pooled sample digestion testing should be performed in combination with a quality assurance system as described by the ICT (http://www.trichinellosis.org/) and in Gajadhar et al. (2019).

The trichinoscope (compression) method, which was used extensively prior to the development of pooled sample digestion methods, is less sensitive and fails to detect non-encapsulated *Trichinella* species. Therefore, trichinoscopy and similar compression methods are not recommended for the examination of food animals and game meats that are intended for human consumption.

### 2.2 Alternative testing methods

Indirect (serological) testing methods are not recommended as a substitute for direct (pooled sample digestion) testing of individual carcasses at slaughter. The ICT has issued recommendations for the use of serological methods in epidemiological studies, surveillance, and certification/verification programs (Gamble et al., 2004; Bruschi et al., 2019).

Direct, molecular-based methods such as PCR are subject to limitations in sensitivity based on the amount of tissue that can be analyzed. Due to the time required to perform these assays and the cost of performing molecular methods, they are not recommended for routine slaughter testing at the present time.

## 3 Post-harvest processing methods

Meat from susceptible animals which might contain *Trichinella* larvae, and which has not been tested as described above, should be further processed to inactivate *Trichinella* larvae prior to distribution for human consumption. An exception is pork that originates from a controlled management compartment that has been certified by the competent authority as having negligible risk for *Trichinella* as described in the *ICT Recommendations on Pre-harvest Control of* Trichinella *in Food Animals* (http://www.trichinellosis.org/Guidelines.html) and in Gamble et al. (2019).

The ICT recognizes three (3) acceptable means of treatment to render potentially *Trichinella*-infected meats safe for consumption: 1) cooking, 2) freezing (for meat from domestic pigs), and 3) irradiation.

### 3.1 Heating/cooking to inactivate *Trichinella* larvae

When the proper equipment is available for accurately achieving and monitoring time and temperature combinations, heating is acceptable for treatment of meat to inactivate *Trichinella* larvae and thereby prevent human trichinellosis (Appendix A). These time/temperature combinations have been demonstrated to be effective for inactivating *Trichinella* in controlled studies (Kotula et al., 1983; FSIS, 2018; European Commission, 2015). Meat that is prepared by heating should be monitored for internal temperature to assure it has reached the minimum temperature throughout.

### 3.2 Freezing to inactivate *Trichinella* larvae

When the proper equipment is available for accurately achieving and monitoring time and temperature combinations, freezing is generally acceptable for treatment of meat from domestic swine to prevent human trichinellosis caused by *Trichinella spiralis* (Kotula et al., 1990; Hill et al., 2009; FSIS, 2018; European Commission, 2015) (Appendix B & Appendix C). Freezing may not eliminate risk posed by species of *Trichinella* that have higher tolerance to cold treatment, including species (*Trichinella nativa*, *Trichinella* genotype T-6) commonly found in wild animals, and freezing, therefore, is not a reliable method for control of *Trichinella* in these animals. Although cold tolerant *Trichinella* spp. have low infectivity for domestic pigs (Pozio et al., 2006), cold tolerance has not been completely defined in certain other species, notably *T. britovi*, which does have significant infectivity for pigs.

### 3.3 Irradiation to inactivate *Trichinella* larvae

The ICT considers irradiation, at levels proven to inactivate *Trichinella* larvae (0.3 kGy), to be an acceptable method for rendering meat safe for human consumption (U.S. Code of Federal Regulations, 1997; Brake et al., 1985). Irradiation is recommended only for sealed packaged foods.

### 3.4 Microwave cooking to inactivate *Trichinella* larvae

Microwave cooking may not result in uniform heating of all areas of meat. Therefore, the desired temperature may be reached in one portion of the meat, whereas another portion of the meat may be undercooked. Studies by Zimmermann and Beach (1982) and Kotula et al. (1982) demonstrated viable *Trichinella* larvae in pork that had been cooked to recommended temperatures or according to manufacturer instructions. Further studies are necessary to consider microwave cooking as a safe option for cooking pork products which may contain *Trichinella*.

### 3.5 Curing to inactivate *Trichinella* larvae

Curing is a preservation process that is widely used in the pork industry to meet certain product characteristics including shelf life, food safety, and palatability/taste. The successful inactivation of *Trichinella* larvae by means of curing depends on the type of raw meat product (semi-dry and dry sausages, ham) as well as the specific processing factors (e.g. salt concentration, ripening conditions) under which the raw meat product undergoes the curing procedure (FSIS, 2018).

There are currently no curing models that can be used to correlate the parameters of meat chemistry (water activity, brine concentration, pH) with time and temperature conditions that can reliably predict inactivation of *Trichinella* spp. (Hill et al., 2017). Therefore, ICT does not recommend curing, salting and smoking processes for control of *Trichinella* in pork, horse or game meats, unless the specific conditions used in the process has been properly validated to be effective.

When curing is used to satisfy food safety requirements, the process used for each specific cured pork meat product must undergo a validation study to prove inactivation of *Trichinella* larvae (using *Trichinella spiralis* as the model species; additional studies using other species of *Trichinella* should provide added security where meat products from pigs or game animals may be at risk of infection with these species). For this purpose, appropriate processing factors (e.g. salt concentration, time period for pickling and ripening) as well as suitable parameters measured in the pork meat product (e.g. salt content and water activity) should be monitored. The efficiency for inactivation of *Trichinella* should be verified by means of bioassay using suitable animal models.

## 4 Consumer education

Consumers should be informed by public health authorities of the risk, and educated in proper meat preparation. Acceptable methods for consumer preparation of meats, which may reduce the public health risk, include:

• Cooking to an internal temperature between 145° F (63 °C) and 160° F (71 °C), followed by a 3-minute rest.
• Freezing of meat from domestic pigs to the minimum times and temperatures listed in the tables of Appendix B, Appendix C. As noted previously, freezing is not recommended for species of *Trichinella* such as *T. nativa* and *T. britovi* (Pozio et al., 2006) and therefore should be used with caution when treating meat from susceptible wild animals, and free-ranging and backyard pigs.

Methods that are not reliable for inactivating *Trichinella* in meats include:

• Cooking using microwaves
• Curing (that has not been validated as described above), salting, drying, or smoking

Education of hunters and others for proper preparation of game meats should follow the same recommendations issued to consumers.

## Conflict of interests

The authors have no conflicts of interest.

## Figures and Tables

**Table A.1:** Required period of heating at temperature indicated.

Minimum internal temperature		Minimum time
Degrees Fahrenheit	Degrees Celsius	
120	49.0	21 h
122	50.0	9.5 h
124	51.1	4.5 h
126	52.2	2 h
128	53.4	1 h
130	54.5	30 min
132	55.6	15 min
134	56.7	6 min
136	57.8	3 min
138	58.9	2 min
140	60.0	1 min
142	61.1	1 min
144	62.2	Instantaneous

(i) All parts of the pork muscle tissue shall be heated according to one of the time and temperature combinations in the table.

(ii) Time and temperature shall be monitored by a calibrated recording instrument.

(iii) The time to raise product temperature from 60 °F to 120 °F (15 °C–49 °C) shall not exceed 2 h unless the product is cured or fermented.

(iv) Time, in combination with temperatures of 138 °F to 143 °F (58.9 °C–61.7 °C), need not be monitored if the product's minimum thickness exceeds 2 in. (5.1 cm) and refrigeration of the product does not begin within 5 min of attaining 138 °F (58.9 °C).

(v) The establishment shall use procedures which insure the proper heating of all parts of the product. It is important that each piece of sausage, each ham, and other product treated by heating in water be kept entirely submerged throughout the heating period; and that the largest pieces in a lot, the innermost links of bunched sausage or other massed articles, and pieces placed in the coolest part of a heating cabinet or compartment or vat be included in the temperature tests.

**Table B.1:** Required period of freezing at temperature indicated.

Temperature °F (°C)	Group 1 (days)	Group 2 (days)
5 (−15.0)	20	30
−10 (−23.3)	10	20
−20 (−28.9)	6	12

(i) Group 1 comprises product in separate pieces not exceeding 6 in. (15 cm) in thickness, or arranged on separate racks with the layers not exceeding 6 in. (15 cm) in depth, or stored in crates or boxes not exceeding 6 in. (15 cm) in depth or stored as solidly frozen blocks not exceeding 6 in. (15 cm) in thickness.

(ii) Group 2 comprises product in pieces, layers, or within containers, the thickness of which exceeds 6 in. (15 cm) but not 27 in. (69 cm), and product in containers including tierces, barrels, kegs, and cartons having a thickness not exceeding 27 in. (69 cm).

(iii) The product undergoing freezing or the containers thereof shall be so spaced to insure free circulation of air between the pieces of meat, layers, blocks, boxes, barrels, and tierces in order that the temperature of the meat throughout will be promptly and uniformly reduced.

(iv) In lieu of the methods prescribed in Table B.1, the treatment of meat may consist of commercial freeze drying or controlled freezing, at the center of the meat pieces, in accordance with the times and temperatures specified in Table C.1 (Appendix C).

(v) The rooms or compartments containing product undergoing freezing shall be equipped with accurate thermometers placed at or above the highest level at which the product undergoing treatment is stored and away from freezer coils.

**Table C.1:** Required period of freezing at temperature indicated.

Minimum internal temperature		Minimum time
Degrees Fahrenheit	Degrees Celsius	
0	−17.8	106 h
−5	−20.6	82 h
−10	−23.3	63 h
−15	−26.1	48 h
−20	−28.9	35 h
−25	−31.7	22 h
−30	−34.5	8 h
−35	−37.2	0.5 h

## Appendix B Methods for the inactivation of *Trichinella* in pork by commercial freezing^1^ (European Commission, 2015; FSIS, 2018; Kotula et al., 1990)

At any stage of preparation, and after chilling to less than 40 °F/5 °C or freezing, all pork muscle or products derived from pork muscle shall be subjected continuously to a temperature not exceeding those specified in Table 1. The duration of freezing at the specified temperature is dependent on the thickness of the meat or inside dimensions of the container.
